# Antimicrobial Profile of Moldovan *Cynara scolymus* L.: Insights into Its Natural Antibiotic Potential

**DOI:** 10.3390/antibiotics14121258

**Published:** 2025-12-12

**Authors:** Cristina Ciobanu, Ludmila Rudi, Laurian Vlase, Greta Balan, Daniela Benedec, Tatiana Calalb

**Affiliations:** 1 Drug Technology Department, Center for Drug Development, Nicolae Testemițanu State University of Medicine and Pharmacy, MD 2004 Chisinau, Moldova; 2 Institute of Microbiology and Biotechnology, Technical University of Moldova, MD 2028 Chisinau, Moldova; 3 Pharmaceutical Technology and Biopharmacy Department, Iuliu Hațieganu University of Medicine and Pharmacy, 400012 Cluj-Napoca, Romania; 4 Microbiology and Immunology Discipline, Department of Preventive Medicine, Nicolae Testemițanu State University of Medicine and Pharmacy, MD 2004 Chisinau, Moldova; 5 Pharmacognosy Department, Iuliu Hațieganu University of Medicine and Pharmacy, 400012 Cluj-Napoca, Romania; 6 Pharmacognosy and Pharmaceutical Botany Department, Center for Drug Development, Nicolae Testemițanu State University of Medicine and Pharmacy, MD 2004 Chisinau, Moldova

**Keywords:** *Cynara scolymus* L., extracts, biological activities, phenolic compounds

## Abstract

**Background**: Artichoke, a medicinal plant with various therapeutic uses, is widely cultivated in many of the world’s geographical areas. The aim of this study was to establish the antimicrobial profile by means of comparative evaluation of the phytochemical constituents, antioxidant, anti-lipid peroxidation, and antimicrobial activities of the basal and cauline leaves, as well as the following by-products: stems, bracts, and inflorescences, from *Cynara scolymus* L. cultivated in the Republic of Moldova. **Methods**: Qualitative and quantitative characterization of the main phenolic compounds from ethanolic extracts was carried out by the HPLC-UV-MS method. The in vitro antioxidant activity was evaluated using DPPH˙, ABTS˙^+^, FRAP, and NO˙ scavenging methods. The lipid-lowering effect was established with a malonic dialdehyde complex and thiobarbituric acid. Antimicrobial properties were screened using the diffusion method. **Results**: The HPLC UV-MS analysis highlighted that the green aerial parts of *C. scolymus* are characterized by the presence of five phenolic acids (kaempferol, gentisic, chlorogenic, *p*-coumaric, ferulic, and caffeic) and four flavonoid heterosides and aglycones (isoquercitrin, quercitrin, luteolin, and apigenin). Correlation between total polyphenolic content and antioxidant activity was found to be statistically significant (*p* < 0.01). The extracts of *C. scolymus*’s aerial parts exhibited significant antibacterial and antifungal activities (*p* < 0.05) against all tested microorganisms, while no inhibitory effect for inflorescences was observed. **Conclusions**: Artichoke leaves and by-products may be considered important and promising sources of bioactive compounds for herbal medicinal products, functional foods, and nutraceuticals, due to their antimicrobial properties. This study makes an original contribution to the specialized literature by the detailed characterization of the antimicrobial profile of the extracts obtained from artichoke cultivated in the Republic of Moldova, a species introduced for the first time on the territory of this country. The obtained results highlight the medicinal potential and economic value of the Moldovan artichoke, with particular emphasis on its by-products: stems, bracts, and inflorescences, which less explored so far, as sources of bioactive compounds. Our analyses indicate that the leaves and by-products of the plant represent promising resources for the development of phytopharmaceutical preparations, functional foods, and nutraceuticals, offering new directions for the integral and sustainable valorization of this species acclimatized in Moldova.

## 1. Introduction

Medicinal plants have been used for thousands of years for health maintenance and remain a primary source of healthcare. Currently, the research of plant extracts with compounds with potential antimicrobial therapeutic applications is an increasingly explored direction in the medical field. This approach constitutes a promising strategy for combating the phenomenon of antibiotic resistance; numerous retrospective studies have highlighted the significant increase in the number of bacterial species that are capable of developing resistance mechanisms to the action of classical antimicrobial agents [1,2]. In this context, many extracts and constituents of plant origin are analyzed to be exploited in the development of new chemotherapeutic applications, with the ability to prevent and treat infections, especially those caused by multidrug-resistant bacteria [3,4,5]. Of particular interest is the species *Cynara scolymus*, which constitutes a gold mine in traditional medicine [6], which gives the *Cynara* species a special importance in research aimed at identifying effective natural alternatives to antibiotics.

Artichoke thistle, *Cynara scolymus* L. (*Cynara cardunculus* var. *scolymus* L.), a species that belongs to the *Asteraceae* family, originally from Ethiopia then spread throughout the Mediterranean basin [7,8], was introduced into culture in temperate areas of Europe, as well as within the experimental collection of the Scientifical Practical Center in the Domain of Medicinal Plants (SPCDMP) (46°56′08.6″ N 28°41′43.4″ E) of the National Institute for Health and Medical Research of Nicolae Testemiţanu State University of Medicine and Pharmacy (Nicolae Testemiţanu SUMPh), from Chisinau, Republic of Moldova. *C. scolymus* is a robust, vivacious plant, perennial in the humid subtropical climate. Temperature is the most important factor in artichoke cultivation; thus, in the temperate areas of Europe, with a mild climate, the plant is grown only by annual cultivation from seeds [9,10]. In addition to temperature, directly proportional to exposure to ultraviolet B rays, is the accumulation of active principles, which are mainly carried out in the cuticle, epidermis, and trichomes [11,12,13].

As mentioned in the literature [14,15] the phytochemical complex of artichoke is formed by groups of substances of secondary metabolism: polyphenols (caffeoylquinic acids—chlorogenic and caffeic acids and cynarin), flavonoids (luteolin and apigenin; flavonosides—rutoside, cynarozide, and scolimoside), sesquiterpene lactones (cynaropicrin), sterol compounds (taraxasterol and pseudotaraxasterol), tannins, and anthocyanins. The diversity of the chemical composition, which possesses a broad spectrum of pharmacological actions such as antioxidant, anti-inflammatory, antibacterial, anti-proliferative, anti-HIV, hepatoprotective, and hypocholesterolemic [16], allows for the use of artichoke as a cholagogue and choleretic [17], hepatoprotective, hypolipidemic, antioxidant, diuretic, hypoglycemic [18], and antimicrobial remedy [19,20,21]. Moreover, artichoke leaf infusion is well-known in folk medicine, traditionally used as a cholagogue and fat metabolism enhancer in the treatment of fever, liver disorders, bile stones, blood cholesterol, urticaria, asthma, and eczema [22,23,24].

The leaf of *C. scolymus*—*Cynarae folium* is recognized as a medicinal plant product in the European Pharmacopeia [25]. Moreover, the Romanian Pharmacopoeia specifies the type of leaves used as basal leaves of the plant [26]. Nevertheless, many phenolic compounds with high antioxidant capacity were found in different parts of artichoke by-products (bract, stem, and inflorescence) [27]. Additionally, El-Nashar et al. demonstrated that the extract obtained from artichoke bract waste exhibits both antioxidant activity and anti-Alzheimer’s potential [28]. Furthermore, Cioni et al. demonstrated that pretreated extracts from artichoke’s stem and bract discards exhibited efficacy against *S. aureus*, *B. cereus* bacteria, and the HSV-2 virus due to metabolites such as cynarine, chlorogenic acid, caffeic acid, luteolin, and apigenin [29]. These bioactive molecules exhibit multiple, often synergistic mechanisms that compromise microbial viability, affecting both Gram-positive and Gram-negative bacteria as well as certain fungi, and can interact with lipid bilayers and membrane proteins through hydrophobic and hydrogen bonding interactions, leading to increased permeability, leakage of ions and cellular contents, and eventual loss of membrane integrity. Pereira et al. demonstrated that phenolic-rich extracts from artichoke leaves caused significant leakage of intracellular nucleic acids and proteins from *Escherichia coli* and *Staphylococcus aureus*, indicating membrane damage as a primary antimicrobial mechanism [30]. Aerial parts of *C. scolymus* are used in the production of nanoparticles through green synthesis [31,32,33]. Sampaio et al. used flower heads to produce silver nanoparticles with antibacterial actions [34]. Khedr et al. compared flower stems and bracts of *C. scolymus* extracts in the green synthesis of silver nanoparticles with apoptotic effects [35]. In the Republic of Moldova, artichoke (*Cynara scolymus* L.) is cultivated and adapted to local pedoclimatic conditions; however, research on this species is very limited [7]. Currently, data on the active principles of *C. scolymus*, as well as the available information regarding its therapeutic properties, remain limited [16,17]. Therefore, this research endeavors to provide an original contribution to the literature data by investigating and defining the extracts obtained from the *C. scolymus* species cultivated in the Republic of Moldova, with particular emphasis on the polyphenolic chemical composition, and some biological properties, especially antimicrobial and antioxidant. The aim is to provide scientific arguments for the use of the leaves, and especially the plant by-products, as promising sources of raw materials for phytopharmaceutical and nutraceutical applications.

## 2. Results

### 2.1. Spectrophotometrical Assays for the Quantification of Total Phenolic Compounds

The results obtained by applying the spectrophotometric methodology allowed for the quantitative estimation of the main groups of chemical compounds from extracts of the aerial parts of *C. scolymus* (basal and cauline leaves, stems, bracts, and inflorescences) cultivated in the collection of the SPCDMP of Nicolae Testemițanu SUMPh. The highest concentrations of total phenolic content were established in basal and cauline leaves. The total polyphenolic amounts in the extracts of aerial parts ranged from 15.47 to 0.94 mg GAE/g dry weight recalculated in a gallic acid equivalent. The total flavonoid content ranged from 7.47 to 0.11 mg/g, expressed as mg of rutin equivalent per gr dry weight. The amounts of phenolic compounds detected in the samples are shown in Table 1.

### 2.2. HPLC-MS Analysis of the Extracts

For a more precise technique, liquid chromatography, with detection by mass spectrometry, was used. *C. scolymus* aerial part extracts were characterized by the presence of ten compounds, including five phenolic acids (kaempferol, gentisic, chlorogenic, *p*-coumaric, and ferulic acids) and five flavonoid glycosides and aglycones (isoquercitrin, myricetin, quercitrin, luteolin, and apigenin), as shown in Table 2.

The analysis highlighted that the compound with the highest concentration proved to be chlorogenic acid in all analyzed extracts, determined to be maximal in basal leaf extract (515.93 µg/mL) and lower in bracts extract (3.98 µg/mL), suggesting that *C. scolymus* plants could serve as a significant source of chlorogenic acid, known for its notable therapeutic benefits.

### 2.3. Antioxidant Properties of C. scolymus Aerial Part Extracts

The antioxidant properties of extracts obtained from leaves, stems, bracts, and inflorescences were determined by applying several specific and non-specific in vitro methods to determine the capacity to capture and neutralize free radicals. Since plant matrices contain chemically different phenolic constituents with distinct electron-donating, radical-scavenging, and metal-chelating capacities, the antioxidant potential of a given extract cannot be fully characterized by a single analytical method. The structural heterogeneity of these compounds requires the application of several complementary tests to obtain a comprehensive and mechanistically meaningful assessment of antioxidant activity.

The scavenging effect of *C. scolymus* extracts, determined by the DPPH˙ method, was measured as the IC_50_ value based on the obtained linear regression graph. The DPPH test revealed statistically significant differences between all tested aerial parts of *C. scolymus* (*p* < 0.001). Basal leaves showed the strongest antioxidant capacity (IC_50_ = 96.07 µg/mL), followed by cauline leaves (125.82 µg/mL). The stems, bracts, and inflorescences showed progressively weaker activity, with the inflorescences having the highest IC_50_ value (6960.92 µg/mL), as shown in Table 3. Tukey’s post hoc test confirmed that all groups differed significantly from each other, forming five distinct statistical groups (a–e). There was a significant correlation between total polyphenolic content with the DPPH˙ scavenging activity of *C. scolymus* aerial part extracts (R^2^ = 0.999, 0.998, 0.998, 0.997, and 0.998, where *p* < 0.01, respectively).

The antioxidant capacity of extracts carried out with ABTS˙^+^, measured as Trolox equivalents, revealed the highest activity for the extract obtained from basal leaves (IC_50_ 32.9 µg/mL) and for the extract obtained from cauline leaves (IC_50_ 29.1 µg/mL). The correlation between total polyphenolic content and the antioxidant test values (ABTS˙^+^ inhibition %) were considered good (R^2^ = 0.9532, 0.9598, 0.916, 0.981, and 0.947, where *p* < 0.01, respectively). Furthermore, the FRAP assay estimated the electron-donating capacity of *C. scolymus*’s aerial part extracts. In this study, the highest FRAP activity of the basal leaf extracts (67.7 μM EDTAE/g dw) was found. Stems and bracts exhibited a lower reduction capacity than *C. scolymus* basal and cauline leaves (*p* < 0.001). The NO˙ radical scavenging assay had the highest significant (*p* < 0.05) NO˙ inhibitory activity among the extracts obtained for basal leaf extract, with a percentage inhibition of 67.7%. The lowest NO˙ radical scavenging percentage was detected in the stem, bract, and inflorescence extracts, with no significant difference (*p* > 0.05).

The results of the antioxidant test for the suppression of LDL oxidation established that 17.5% of lipoproteins are oxidized in the absence of copper sulfate, the negative control sample (Cu^−^), under conditions of 37 °C for 24 h. The highest activity in counteracting LDL oxidation was established for the samples with the application of leaf extracts (60.8–61.2% inhibition), with no significant difference (*p* > 0.05). For the extracts obtained from artichoke bracts and stems, the malonic dialdehyde test values were57.82% and 54.13%, respectively (*p* < 0.05). The inflorescence extract did not show antioxidant effects under conditions of induced lipoprotein oxidation. Ascorbic acid, in the 1 mg/mL concentration, maintained 58.2% of the experimental lipoproteins in the non-oxidized form. The antioxidant activity of 1 mg of *C. scolymus* extract, which determines its ability to suppress lipoprotein oxidation, is similar to the activity of 1 mg of ascorbic acid. Therefore, the antioxidant compounds contained in *C. scolymus*’s aerial part extracts possess the ability to counteract the oxidation of low-density lipoproteins, and the antioxidant effect is achieved based on the mechanism of proton and electron transfer.

The use of these multiple methods provides a robust and reliable assessment of the extracts’ antioxidant potential. These statistically significant differences suggest that the phenolic compounds present in artichoke exhibit distinct antioxidant behaviors, likely reflecting their diverse structures and position of hydroxyl groups, degree of conjugation, steric accessibility, solubility, and mechanisms of action.

### 2.4. Antimicrobial Activity of C. scolymus Aerial Part Extracts

The antibacterial and anti-candida activities of *C. scolymus* samples were evaluated against some bacterial strains, such as *Staphylococcus aureus* derived from ATCC^®^ 25923, *Corynebacterium diphtheriae* ATCC^®^ 13812, Bacillus cereus ATCC 11778, *Enterococcus faecalis derived from ATCC^®^* 19433, *Escherichia coli* derived from *ATCC^®^* 25922, and *Pseudomonas aeruginosa* derived from ATCC^®^ 27853, but also against a fungus, *Candida albicans* (ATCC^®^ 0231). As shown in Table 4, the results showed that the green aerial plant extracts of *C. scolymus* efficiently suppress the growth of microorganisms, with variable efficacy, although with significantly lower potency (*p* < 0.05) compared to the positive controls (tetracycline and miconazole).

Most bacteria were susceptible to *C. scolymus* basal and cauline leaf extracts, whereas *S. aureus*, *E. coli*, *C. diphtheriae*, and *B. cereus* were most sensitive, as demonstrated by low minimum inhibitor concentration (MIC) values. The results of the antimicrobial activity of the four aerial part extracts (basal and cauline leaves, stems, and bracts) suggested that *P. aeruginosa* was the most resistant strain to the analyzed plant extracts, followed by *E. faecalis*. Furthermore, the extract obtained from the *C. scolymus* inflorescence did not show antibacterial or antifungal actions in the investigated concentrations. The antifungal activity of basal leaves extract against *C. albicans* started at 1.466 mg/mL with an inhibition zone of 8.1 mm, cauline leaves at 1.532 mg/mL with an inhibition zone of 7.7 mm, and bracts extract at 1.635 mg/mL with an inhibition zone of 6.2 mm, stems extract suppressed yeast grow at a concentration of 3.435 mg/mL with an inhibition zone of 7.25 mm, respectively.

### 2.5. Time-Kill Kinetics

The time-kill kinetic profiles of extracts also showed different characteristics depending on the microbial species.

*S. aureus* exposed to basal leaf extract showed a rapid decrease in viable cell counts during the first 12 h at both ×1 MIC and ×2 MIC. At ×2 MIC, the decline continued through the 24 h mark. The killing kinetics demonstrated a concentration-dependent anti-*S. aureus* effect of basal leaves extract, exhibiting bacteriostatic activity at ×1 MIC and bactericidal activity at ×2 MIC. A rapid reduction in bacterial cell counts was observed against *E. coli* during the first 6 h at ×2 MIC and the first 12 h at ×1 MIC. At ×2 MIC, this decrease continued gradually throughout the 24 h incubation period. The kill-rate curve against *C. albicans* showed a concentration-dependent decrease in viable cell counts during the first 24 h of exposure at both ×1 MIC and ×2 MIC, after which the number of viable cells remained stable up to 48 h.

For cauline leaf extract against *S. aureus* and *E. coli*, the kill-rate curves showed a concentration-dependent reduction in bacterial cell counts during the first 6 h at both ×1 MIC and ×2 MIC, followed by stabilization through the 24 h incubation period. Similarly, for cauline leaves against *C. albicans*, the kill-rate curves demonstrated a concentration-dependent reduction in viable cell counts during the first 24 h at both ×1 MIC and ×2 MIC, followed by slight regrowth up to 48 h of incubation.

The time-kill kinetics profile of plant stem extract against *S. aureus* revealed a decrease in viable cell counts at 5 h of incubation at both ×1 MIC and ×2 MIC, followed by stabilization of the population up to 24 h. For *E. coli*, a reduction in viable cell counts was observed at 12 h at both concentrations. The time-kill kinetics profile against *C. albicans* demonstrated concentration-dependent activity at all concentrations tested. At ×2 MIC, the viable cell count dropped sharply within the first 6 h, followed by a slower decline up to 12 h and another pronounced decrease at 24 and 36 h. At ×1 MIC, a marked reduction in cell count was observed at 36 and 48 h.

For the artichoke bracts extract, the time-kill kinetics profiles showed species-dependent responses. Against *S. aureus*, a reduction in viable cell counts was observed at 6 h at ×2 MIC and at 12 h at ×1 MIC. For *E. coli*, both concentrations produced a decrease in cell counts at 6 h. In the case of *C. albicans*, a decline was noted at 6 h, followed by a slow regrowth phase that continued to 48 h (see Supplementary Materials).

## 3. Discussion

The present study brings novelty by researching phytochemical content and determining the antioxidant, LDL peroxidative, and antimicrobial actions of both artichoke leaves and artichoke by-products (bracts, stems, and inflorescences), which demonstrate the possibility to encompass the entire aerial part of the plant in natural products processing. The phenolic compounds in *C. scolymus* have been widely studied due to the varied therapeutic potential [36,37]. Their presence and abundance are related to metabolic reactions, which are influenced by the analyzed botanical part, the ontomorphogenetic phase of plant development, and climatic growing conditions, including the complex of abiotic stressors [38]. Phenolic compounds are polar compounds [39]; thus, for their extraction, ethanol of 70% was used, based on previous research [40]. The spectrophotometric assay regarding the amount of secondary metabolites of phenolic nature in artichoke leaf, showed that the total polyphenolic content is higher than the total flavonoid content, similar to other authors [41,42]. In Romanian artichoke leaf extract, obtained through maceration with water, the total phenolic content (TPC) was quantified as 15.2 mg/g [43]. Studies analyzing artichoke leaves from Poland reported TPC values of 33.5 mg/g and a total flavonoid content (TFC) of 17.9 mg/g for methanolic extracts [44], and a TPC of 27 mg/g for ethanolic extracts [45]. The variation in total phenolic content across the literature could be explained by both extrinsic and intrinsic factors, including the solubility of the compounds in the solvents used. This solubility is influenced by the structure of the hydroxyl groups and the molecular size and length of the hydrocarbon chains of the bioactive compounds [46]. Artichoke by-products, generated during agricultural procedures and the processing industry, represent a significant amount of discarded material. Complementary studies conducted in other cultivation areas on *C. scolymus* agro-industrial discards, such as stems, bracts, and inflorescence, show variability in the content of total polyphenols and flavonoids. The TPC of Moldovan artichoke discards, compared to the optimized ethanolic extract of bracts and stems from Portugal [47] (where the TPC was 21.6 mg/g) and to Italian artichoke bract extracts (5 mg/g) [48,49], is lower. Nevertheless, the content is higher compared to Turkish methanolic extracts (2.4 mg/g) [50].

Further, through HPLC-MS investigation, we revealed that the leaves, stem, bracts, and inflorescences contain hydroxycinnamic acid derivatives such as chlorogenic and caffeic acids but also two flavonoid aglycones (apigenin and luteolin), these active principles are responsible for the health-promoting effects, especially through the antibacterial, antifungal, antioxidant, anti-inflammatory, hypolipidemic, and antiatherogenic activities recorded in the literature data [19,29,51]. The results obtained for quantifying phenolic metabolites by HPLC-UV-MS analysis indicated chlorogenic acid, the main bioactive compound. Caffeic acid was also found in high amounts in our studied aerial part samples, followed by the aforementioned flavonoids, apigenin, luteolin, and hydroxycinnamic acid derivatives *p*-cumaric and ferulic acids. Our investigation confirms the presence of phenolic compounds reported in previous publications [21,52,53], along with distinctions [54], continuing to support phytochemical diversity and therapeutic potential of aerial part extracts.

The antioxidant potential of *C. scolymus*’s aerial parts was thoroughly assessed using DPPH˙, ABTS˙^+^, FRAP, and NO˙ in vitro methods, affirming the promising role of the green aerial parts of the plant as natural antioxidants. Basal and cauline leaves of *C. scolymus* grown in the steppe climate conditions of the Republic of Moldova highlighted remarkable DPPH˙ and ABTS˙^+^ radical scavenging activity, an effect attributed to their high content of chlorogenic acid. Therefore, the results of the positive correlation between the TPC, TFC, and the in vitro DPPH˙ and ABTS˙^+^ antioxidant methods highlight that the active polyphenolic principles, such as electron or hydrogen donors and singlet oxygen quenching, demonstrate the important antiradical activity of *C. scolymus* green aerial parts, which has been recorded by other authors as well [55,56].

Moreover, the electron-donating capacity of *C. scolymus* aerial part extracts was monitored by FRAP assay. This assay relies on the reduction in the ferric–TPTZ complex to its ferrous, intensely blue form (Fe^2+^–TPTZ) by antioxidant polyphenols [57]. The absorbance of this resultant blue-green colored solution of samples was measured at 700 nm, which was related to the Fe^2+^ amount in the mixture. The ability to reduce the ferric ions (Fe^3+^) was recorded for basal leaves, cauline leaves, stems, and bracts. The FRAP assay confirmed the reducing power in the limits of 67.7 μM EDTAE/g dw to 22.45 μM EDTAE/g dw.

Aerial parts of *C. scolymus* were examined for their possible scavenging ability of NO˙. The principle of the method consists of determining the production of the nitric oxide radical generated by sodium nitroprusside. Nitric oxide interacts with oxygen and forms nitrites, which are determined spectrophotometrically using the Greiss reagent. The chromophore formation occurs due to the diazotization of nitrite with sulfanilamide and its coupling with naphthyl ethylenediamine [58]. The highest inhibitory activity among the extracts was obtained for basal leaves extract with a percentage inhibition of 67.7%; the lowest NO˙ radical scavenging activity was detected in stems, bracts, and inflorescence extracts. The results of the antioxidant activity of the aerial parts of *C. scolymus* assessed by these in vitro assays offer reliable insights into the intrinsic radical scavenging potential with minimal external influence. It is well known that polyphenolic compounds exhibit various pharmacological activities, including hypolipidemic and antiatherogenic effects [59]. Mocelin et al. revealed a significant decrease in oxidized LDL concentration and antioxidized LDL in rats treated with the leaf extract of *C. scolymus* [51]. Furthermore, Mokhtari et al. demonstrated a significant improvement in plasma lipid profiles by reducing total cholesterol, triglycerides, and LDL–cholesterol while increasing HDL–cholesterol during the administration of artichoke bract extract in mice [60]. In our study, we showed that the green aerial parts of *C. scolymus* have the ability to suppress human low-density lipoprotein oxidation in vitro. The LDL was greatly reduced by basal and cauline leaf extracts (61.2% and 60.8%), followed by bract extracts (57.82%). The lowest percentage of inhibition of LDL oxidation was observed for artichoke stem extract (54.13%). The inflorescence did not exhibit an antioxidant effect under conditions of induced lipoprotein oxidation. Our results suggest that the LDL oxidation capacity is possible due to myricetin, quercetin, isoquercitrin, and kaempferol, which are reported in the literature data as exhibiting favorable hypolipidemic effects [61] and identified in *C. scolymus* basal and cauline leaves, stems, and bracts but not in inflorescences.

To assess the antimicrobial profile of the aerial parts of *C. scolymus* cultivated in the Republic of Moldova, we determined the antimicrobial activity of the extracts in the study. The antimicrobial properties of *C. scolymus* extracts, cultivated worldwide and reported throughout the years, have been commonly associated with secondary metabolites such as flavonoids, tannins, essential oils, glycosides, and phenols [62,63,64]. There is a wide range of results, mostly varying depending on the area from where the artichoke was harvested and the extraction method, though not always [54]. The antimicrobial assay carried out by Scavo et al. showed that *C. cardunculus* L. var. *altilis* ethanolic extract was found to be the most active and effective in inhibiting the growth of Gram-positive species [65]. Zhu et al. revealed that leaf extract was found to be the most effective against all of the tested organisms, followed by the artichoke head and stem extracts, and the ethanol fraction showed the most significant antimicrobial activity compared to other extraction solvents [66]. The antimicrobial activity of the plant discards shows that Moldovan artichoke inflorescence was ineffective against strains used in the experiments, unlike Mejri et al.’s inflorescence extract, which did show antimicrobial activity against *S. aureus*. But the lack of inhibitory effect against *E. coli* and *C. albicans* confirms the attribution of antibacterial activity mainly to hydroxycinnamic acids and flavones [67]. Our results of the antimicrobial assay showed important antimicrobial potential for basal and cauline leaves, stems, and bracts’ ethanolic extracts, against the bacterial strains and fungi chosen in this study: *S. aureus*, *B. cereus*, *C. diphtheriae*, *E. faecalis*, *P. aeruginosa*, *E. coli*, and *C. albicans*. These outcomes can be aligned with the antioxidant assay results, supporting the hypothesis that biological activity is closely linked to the polyphenol composition of the extracts. The MIC values established for artichoke by-product extracts were consistently lower for Gram-positive bacteria, indicating a higher susceptibility compared to Gram-negative strains. This trend is in line with structural differences between the two groups, as the thick peptidoglycan layer of Gram-positive bacteria is generally more accessible to phenolic compounds. Nevertheless, a noteworthy aspect of the antibacterial profile of *C. scolymus* L. is its inhibitory activity against *P. aeruginosa*, a pathogen well known for its high level of intrinsic resistance. This inhibitory effect can be attributed particularly to chlorogenic acid and luteolin derivatives, which are expected to interfere with membrane integrity, quorum-sensing pathways, and biofilm formation in *P. aeruginosa* [30]. These data are of paramount importance for medicine and healthcare in order to diminish the burden of antimicrobial resistance by subsequently using *C. scolymus* standardized extracts as alternative antimicrobial drugs. In addition, time-kill studies offer a more comprehensive understanding of the pharmacodynamics of a potential antibacterial agent compared to endpoint measurements such as MIC. Therefore, time-kill assays are essential for quantifying pharmacodynamic effects by measuring the reduction in bacterial growth over time in relation to drug concentration [68]. In our study, across all experiments, the time-kill kinetics of the extracts consistently demonstrated species- and concentration-dependent effects.

## 4. Materials and Methods

### 4.1. Chemicals

The 18 reference substances used for the HPLC-UV-MS method were provided by the Sigma-Aldrich Company (Schnelldorf, Germany): caftaric (>97%), vanillic (≥97%) *p*-coumaric (≥98%), ferulic (≥99%), chlorogenic (>95%), sinapic (≥98%), caffeic (≥95%), and gentisic acids (>95%), as well as the following flavonoids: isoquercitrin (≥98%), quercitrin (≥78%), fisetin (≥98%), patuletin (≥98%), apigenin (>95%), luteolin (≥98%), myricetin (≥97%), hyperoside (≥95%), kaempferol (≥97.0%), and rutin (≥94%), with chromatographically pure reagents. From Sigma-Aldrich (Schnelldorf, Germany) galic acid (≥98.0%), DPPH˙, FRAP, Folin–Ciocalteau and Greiss reagents, sodium nitroprusside dihydrate, and Trolox (>97%) were also obtained. Ascorbic acid and potassium persulfate were obtained from Merck (Darmstadt, Germany). ABTS˙^+^ was obtained from Alfa Aesar GmbH & KG (Karlsruhe, Germany). EDTA and TPTZ were purchased from HiMedia Laboratories (Thane, India). All solvents and chemical reagents used were of analytical grade or higher.

### 4.2. Plant Materials

**Figure 1:**
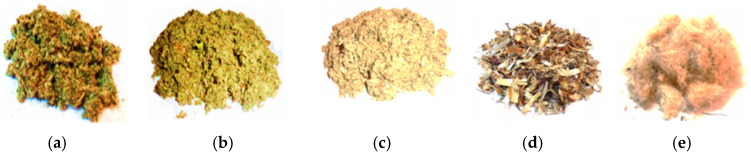
Grinded aerial parts of *C. scolymus* used in the experimental analyses: (**a**) basal leaves; (**b**) cauline leaves; (**c**) stems; (**d**) bracts; (**e**) inflorescences.

Specimens of *Cynara scolymus* L. aerial parts, basal leaves, cauline leaves, stems, bracts, and inflorescences, were collected from the Scientifical Practical Center in the Domain of Medicinal Plants of Nicolae Testemițanu SUMPh from Chisinau, Republic of Moldova, during the flowering period (July 2024). The taxonomic affiliation of artichoke thistle to the *C. scolymus* species was determined and confirmed by macro- and microscopic studies. Labeled, naturally dried samples of *C. scolymus* species collected in the experimental collection of SPCDMP were kept in the Herbarium at Pharmacognosy and Pharmaceutical Botany Department of the Faculty of Pharmacy of *Nicolae Testemițanu* SUMPh, with the voucher code (CS).2004.2.24. The harvested aerial parts of *C. scolymus* (Figure 1) were ground into powder using a RETSCH laboratory knife mill at 5000 rpm and after pulverization, the samples were sieved through a 0.8 mm mesh.

### 4.3. Extract Preparation

To obtain the plant extracts, the Soxhlet extraction method assisted by ultrasound was used. Ten grams of powdered plant materials was extracted with 70% ethanol, the vegetable matter-to-solvent ratio was 5:100 (*w*/*v*). This extraction was performed for 4 h at the boiling temperature of the solvent [69]. The dried extracts were obtained by filtration and evaporation to dryness on a Laborota 1011 Evaporator. The extraction yields (Y %) of *C. scolymus* basal leaves, cauline leaves, stems, bracts, and inflorescences were determined using the following relation: Y (%) = (m_e_∙100)/m_p_, where m_e_: mass of the dry extract obtained (g); m_p_: mass of dried plant product (g).

### 4.4. Total Poyphenolic Content (TPC) Assessment

Each extract’s total polyphenolic content (TPC) was measured using a modified Folin–Ciocalteu procedure based on the Singleton method [70]. A volume of 150 μL Folin–Ciocalteu reagent (1/10) was added to 300 μL of the extract sample. After incubation for 10 min at room temperature, 1.2 mL of 10% sodium bicarbonate solution and 1.35 mL of purified water was added. The samples were stored for 45 min in the absence of light [69]. The absorbance of the samples was determined at 765 nm using a Specord 200 Plus Spectrofotometer (Analytik Jena, Hallertau, Germany) against a blank solution. A calibration curve was established by employing standard gallic acid (Sigma Aldrich), encompassing concentrations ranging from 0 to 115 μg/mL (y = 0.1119x − 0.0025; R^2^ = 0.9993). The outcomes were expressed in milligrams of gallic acid equivalents (GAEs) per gram of dry weight (dw).

### 4.5. Total Flavonoid Content Assessment

The flavonoid concentration of each sample was assessed spectrophotometrically with aluminum chloride serving as the color reagent [69]. Each plant extract was dissolved in methanol and mixed with 0.1 mL of 10% aluminum chloride hexahydrate, 0.1 mL of 1 M potassium acetate, and 2.8 mL of deionized water. After incubation for 40 min at room temperature, the absorbance was recorded at 415 nm. Rutin, in the range 0.05–0.1 mg/mL, served as the standard (y = 29.116x − 0.0181; R^2^ = 0.9992). Results are expressed as rutin equivalents (mg RE/g dry weight).

### 4.6. Analyses Using High-Performance Liquid Chromatography

HPLC coupled with an HP 1100 autosampler, HP 1100 thermostat, HP 1100 UV detector, and Agilent Ion Trap 1100 VL mass spectrometer was used. The working conditions were as follows: analytical column—Zorbax SB-C18 100 mm × 3.0 mm, 3.5 µm; Zorbax SB-C18 precolumn (Agilent Technologies, Santa Clara, CA, USA); mobile phase: methanol mixture–acetic acid solution 0.1% (*v*/*v*), gradient elution (start 5% methanol, up to 35 min 42% methanol, up to 38 min 42% methanol, up to 45 min 5% methanol—reequilibration); flow rate: 1 mL/min; temperature: 48 °C; detection: ultraviolet, 330 nm up to 17 min, 370 nm up to 38 min/MS; injection volume: 5 µL. MS working conditions were as follows: ion source: ESI (electrospray); ionization mode: negative; nebulizer: nitrogen, pressure 70 psi; drying gas: nitrogen, flow rate 12 L/min; temperature 360 °C; capillary potential: +3000 V; analysis mode: specific ion monitoring (polyphenolcarboxylic acids) or auto MS (flavonoids and their aglycones). Each class of compounds was detected at the wavelength corresponding to the maximum absorption of the UV spectrum. For quantitative determination, a calibration graph was made for each compound in the concentration range 0.5–5.0 µg/mL. Two types of samples of each aerial part extracts were analyzed in parallel, one as such, and the other hydrolyzed. The reason that hydrolysis was performed is that, usually, some flavone aglycones or some polyphenol carboxylic acids are not in a free state, but bound as glycosides and esters, etc. [71]. Hydrolysis was performed according to the following protocol: one part of the extract was diluted with one part of 2 N hydrochloric acid solution and maintained on a water bath at 80 °C for 60 min.

### 4.7. Antioxidant Activity Assays

#### 4.7.1. DPPH Free Radical Scavenging Assay

The antioxidant activity was evaluated using the DPPH˙ test [69]. A stock solution of 24 mg DPPH˙ in 100 mL methanol was prepared and stored at 20° C. The working solution was diluted to an absorbance of ~0.98 ± 0.02 at 517 nm (UV–Vis Jasco V530, Jasco, Tokyo, Japan). An aliquot of 3 mL of the working solution was combined with 100 μL of sample (10–500 μg/mL), mixed thoroughly, and incubated for 15 min. Absorbance was measured at 517 nm. Controls (c) contained all reagents except the samples (s). DPPH˙ scavenging activity (%) was calculated as follows: ${\mathrm{AA}}_{\mathrm{DPPH}˙}(\%)=\frac{A_{c}\;-\;A_{s}}{A_{c}}\times 100$. The IC_50_, the sample concentration scavenging 50% of DPPH˙ radicals, was determined from a plot of scavenging activity (%) versus concentration.

#### 4.7.2. ABTS Assay

2,2-azinobis (3-ethylbenzthiazoline-6-sulphonic acid), commonly called ABTS˙^+^, cation scavenging activity was performed [72]. ABTS˙^+^ radicals were prepared by reacting 7 mM ABTS with 2.45 mM potassium persulfate, incubated for at least 16 h. The solution was mixed with methanol (50%) to an absorbance of ~0.70 ± 0.02 at 745 nm (30° C). Antioxidant activity was measured by mixing 300 μL of extract with 3.0 mL of ABTS˙^+^ solution, and IC_50_ values were calculated as the concentration required for 50% radical reduction.

#### 4.7.3. Assessment of Ferric-Reducing Antioxidant Power (FRAP)

FRAP was determined following Benzie and Strain [73]. The FRAP reagent was prepared by combining 25 mL acetate buffer (30 mM, pH 3.6), 2.5 mL TPTZ (10 mM), and 2.5 mL ferric chloride (20 mM) and was incubated at 37 °C for 15 min. EDTA (50–500 mg/L) served as the standard and results were expressed as EDTA equivalents (μg EDTAE)/g dw), with higher values reflecting stronger antioxidant activity.

#### 4.7.4. Nitric Oxide-Reducing Assay

Immediately before the assay, 10 mM sodium nitroprusside was dissolved in 20 mM phosphate buffer (pH 7.4) to prepare the working solution. The reagent mixture contains 0.5 mL of the sample and 0.5 mL of the sodium nitroprusside solution and is incubated at 25 °C for 150 min. After incubation, 2 mL of Greiss reagent (1% sulfanilamide solution, 2% phosphoric acid solution, and 0.1% naphthylethylenediamine dihydrochloride solution) is added to the reagent mixture and the absorbance is measured at a wavelength of 546 nm [74]. Ascorbic acid was used as the positive control at a concentration of 0.1 mg/mL.

#### 4.7.5. In Vitro Determination of the Capacity to Inhibit Low-Density Lipoprotein Oxidation

The LDL oxidation assay, approved by the ethics committee of the Institute of Microbiology and Biotechnology from the Republic of Moldova, was performed using human LDL isolated from blood serum by the heparin–manganese precipitation method, according to established procedures [72,73,75]. Briefly, 2 mL of serum was mixed with 400 EU heparin and 150 μL of 1 M MnCl_2_, incubated for 30 min at 0 °C, and centrifuged under the same conditions. The resulting pellet was washed with 0.9% NaCl and recentrifuged, after which the lipoprotein fraction was resuspended in 1 M NaCl to obtain a final protein concentration of 2 g/L. For the oxidation assay, 0.1 mL LDL solution was combined with 10 μL of extract and incubated for 5 min. Oxidation was initiated by adding 33.3 μL of 50 μM CuSO_4_, followed by incubation for 24 h at 37 °C. The reaction was terminated using EDTA (final concentration 27 mM). LDL oxidation was quantified by determining thiobarbituric acid-reactive substances (TBARS), as described in references [76,77]. Samples were mixed with 1 mL of 0.67% TBA and 1 mL of 15% TCA, heated for 1 h at 95 °C, cooled, and centrifuged at 3000× *g* for 15 min. Absorbance of the MDA–TBA complex was measured at 535 nm using a T80+ UV/Vis spectrophotometer (PG Instruments Ltd., Wibtoft, UK). The results were calculated based on protein content and expressed as the percnetage of inhibition relative to the oxidized LDL control. Ascorbic acid (0.1 mg/mL) served as a positive control, while a negative control without CuSO_4_ was included to exclude LDL autoxidation.

### 4.8. Antimicrobial Activity

For the bioassay, six bacterial strains, *Staphylococcus aureus* (ATCC 25923), *Escherichia coli* (ATCC 25922), *Pseudomonas aeruginosa* (ATCC 27853), *Bacillus cereus* (ATCC 11778), *Corynebacterium diphtheriae* (ATCC 13812), *Enterococcus faecalis* (ATCC 19433), and one fungal strain of *Candida albicans* (ATCC 10231), were taken into account. The antimicrobial activity was assessed by the agar-well diffusion method [78,79]. Wells (6 mm) were filled with 100 µL of plant extract on Mueller–Hinton agar inoculated with microbial suspensions (0.5 McFarland). Plates were refrigerated for 30 min for diffusion and incubated at 37 °C for 18 h. Inhibition zones, including the well diameter, were measured to determine activity. The MIC (minimal inhibitory concentration) and MBC (minimal bactericidal concentration)/MFC (minimal fungicidal concentration) values of *C. scolymus* samples were assessed using the broth dilution method in accordance with CLSI (USA) guidelines [80]. Serial two-fold dilutions of samples were prepared in broth with bacterial suspensions adjusted to 10^8^ CFU/mL (0.5 McFarland standard) to determine the MIC. Controls included inoculated broth (growth control) and broth with extract only (sterility control). The MIC was defined as the lowest concentration showing no visible growth after 24 h incubation at 37 °C and expressed in mg/mL. MBC/MFC was defined as the lowest concentration, killing ≥99.9% of the initial inoculum. Tetracycline (10 µg/mL) and miconazole (10 µg/mL) were used as positive controls for bacteria and fungi, respectively. All assays were performed in triplicate.

### 4.9. Time-Kill Kinetics Assay

The time-kill experiment was performed as described in the literature [68]. Three species were tested: *S. aureus* ATCC 25923 (Gram-positive), *E. coli* ATCC 25922 (Gram-negative), and *C. albicans* ATCC 10231 (fungus). An 18–24 h bacterial culture was used to prepare a suspension in sterile saline solution, resulting in a final inoculum of 1 × 10^6^ CFU/mL. Plant extracts were added to the bacterial suspension at final concentrations corresponding to 0.5, 1, and 2 × MIC. The mixture was then incubated at 37 °C. At predetermined time points, 0, 1, 2, 3, 4, 5, 6, 12, and 24 h, for bacteria, and 0, 6, 12, 24, and 36 h for fungi, 100 µL from each tube was plated on Mueller–Hinton agar. Plates were incubated at 37 °C for 24–36 h, after which the number of colonies (CFU/plate) was counted and converted to CFU/mL. Mueller–Hinton Broth (MHB) without extract and MHB containing tetracycline/miconazole were used as controls, as presented in Supplementary Materials. All experiments were performed in triplicate.

### 4.10. Statistical Methods

Correlations among the measured and derived traits were evaluated by calculating Pearson correlation coefficients using Microsoft Excel 2022. Data were analyzed by one-way analysis of variance (ANOVA) using SPSS software version 20.0 (IBM Corporation, Chicago, IL, USA). Differences were considered statistically significant at *p* < 0.05, and all results are presented as mean ± standard deviation (SD).

## 5. Conclusions

This study focused on the determination of the antimicrobial profile of basal and cauline leaves of *C. scolymus* as well as its stems, bracts, and inflorescence by-products and identifying traceability through their chemical composition and antioxidant properties. According to the HPLC-UV-MS analysis, the ethanolic extracts investigated have the key compounds chlorogenic and caffeic acids, luteolin-7-O-glucoside and apigenin, with organ-specific variations in concentration. Overall antioxidant capacity, assessed in vitro through DPPH˙, ABTS˙^+^, FRAP, and NO˙ assays, demonstrated high scavenging potential of the green aerial parts of the species. Specifically, we reveal strong positive correlations between TPC and antioxidant capacity, further validating the contribution of these compounds to the biological activity of *C. scolymus*. The results of the antimicrobial assay showed important antimicrobial potential for the leaves and the green by-products of *C. scolymus* with varying degrees of potency. The most notable effect was observed against Gram-positive bacteria, including *Staphylococcus aureus*, *Corynebacterium diphtheriae*, *Bacillus cereus*, and *Enterococcus faecalis*, as well as antifungal activity. Time-kill kinetics analysis indicated that the antibacterial effects of the extracts were either concentration-dependent or time-dependent, varying with both the type of extract and the microbial strain. Thus, *C. scolymus* leaves and by-product extracts may represent a more sustainable pathway toward achieving the goals of One Health in an age increasingly threatened by antibiotic resistance.

## Figures and Tables

**Table 1:** Extraction yield, total polyhenolic, and flavonoidic values of the aerial parts extracts of *C. scolymus*.

Samples	Yield (%)	TPC (mg/g dw GAE)	TFC (mg/g dw RE)
Basal leaves	17.24	15.47 ± 0.86	7.47 ± 1.32
Cauline leaves	17.14	13.18 ± 0.73	5.84 ± 0.66
Stems	13.12	6.62 ± 0.39	1.95 ± 0.92
Bracts	14.96	2.56 ± 0.40	1.39 ± 0.37
Inflorescences	3.88	0.94 ± 0.44	0.11 ± 0.08

Values are expressed as the mean of 3 determinations ± SD, (*p* < 0.01); dw: dry weight.

**Table 2:** Phenolic compounds identified in *C. scolymus* extracts by HPLC-UV-MS.

Polyphenolic Compounds	RT ± SD (min)	[M-H]- Exp. (*m*/*z*)	Basal leaves (µg/mL)	Cauline leaves (µg/mL)	Stems (µg/mL)	Bracts (µg/mL)	Inflorescences (µg/mL)
Gentisic acid	2.15 ± 0.07	179	BLQ	BLQ	BLQ	BLQ	ND
Caffeic acid	5.60 ± 0.04	173	138.944 ± 0.79	123.469 ± 0.654	11.031 ± 0.253	4.202 ± 1.085	0.190 ± 0.216
Myricetin	21.13 ± 0.06	179	BLQ	BLQ	BLQ	BLQ	ND
Quercitrin	23.00 ± 0.13	447	BLQ	BLQ	BLQ	BLQ	ND
Luteolin-7-O-glucoside	29.10 ± 0.19	285	74.981 ± 0.184	24.411 ± 0.356	2.289 ± 0.332	1.897 ± 0.036	0.673 ± 0.077
Kaempferol	31.60 ± 0.17	595	BLQ	BLQ	BLQ	BLQ	BLQ
Apigenin	33.10 ± 0.15	269	13.791 ± 0.723	23.179 ± 1.73	2.201 ± 0.22	3.991 ± 0.2	4.740 ± 0.24
Chlorogenic acid	5.62 ± 0.05	353	515.93 ± 8.966	485.74 ± 9.097	115.07 ± 6.679	3.98 ± 0.301	12.25 ± 0.488
*p*-coumaric acid	8.7 ± 0.08	163	1.397 ± 0.019	1.255 ± 0.07	0,292 ± 0,07	0,419 ± 0,024	ND
Ferulic acid	12.2 ± 0.10	193	1.495 ± 0.028	0.789 ± 0.028	0.313 ± 0.04	0.749 ± 0.035	ND
Izoquercitrin	19.60 ± 0.10	463	BLQ	BLQ	BLQ	BLQ	ND

Values are the mean ± SD (*n* = 3). BLQ—below limit of quantification (0.1 µg/mL); ND: not detected compound.

**Table 3:** Antioxidant capacity of vegetative aerial parts of *C. scolymus*.

Samples	DPPH˙ IC_50_ (µg/mL)	ABTS˙^+^ IC_50 (_µg/mL)	FRAP (μM/g dw)	NO˙ I %	LDL Oxidation I %
Basal leaves	96.14 ± 0.17	29.1 ± 0.37	67.7 ± 0.7	60.1 ± 0.12	61.2 ± 0.40
Cauline leaves	125.82 ± 0.22	32.9 ± 0.23	56.97 ± 1.31	57.52 ± 0.13	60.8 ± 0.38
Stems	412.89 ± 0.48	80.03 ± 1.17	33.58 ± 0.39	50.27 ± 0.06	54.13 ± 0.87
Bracts	2182.68 ± 0.65	1446 ± 1.55	22.45 ± 0.32	50.18 ± 0.003	57.82 ± 0.39
Inflorescences	6960.92 ± 0.21	1011.39 ± 1.07	N/E	50.45 ± 0.05	N/E
Trolox	12.08 ± 0.03	2.55 ± 0.08	-	-	-
EDTA	-	-	99.58 ± 0.01	-	-
Ascorbic acid	-	-	-	85.7 ± 0.05	58.2 ± 0.01

Each value is the mean ± SD of three independent measurements. N/E—no effect.

**Table 4:** Antimicrobial and antifungal activity of *C. scolymus*’ aerial parts against bacteria and yeast strains.

Test Strains	Zone of Inhibition, (mm)							MIC, (mg/mL)							MBC/MFC, (mg/mL)						
	BL	CL	ST	BC	IF	TC	MC	BL	CL	ST	BC	IF	TC	MC	BL	CL	ST	BC	IF	TC	MC
*B. cereus*	10.2 ± 0.20	9.3 ± 0.58	9.2 ± 0.7	8.1 ± 0.10	N/E	21.0 ± 1.00	N/A	0.301 ± 0.03	0.259 ± 0.05	0.344 ± 0.02	0.448 ± 0.03	N/E	0.001 ± 0.00	N/A	0.301 ± 0.03	0.592 ± 0.06	0.793 ± 0.01	0.879 ± 0.06	N/E	0.001 ± 0.00	N/A
*C. diphtheriae*	12.4 ± 0.47	11.1 ± 0.40	6.2 ± 0.20	7.2 ± 0.20	N/E	22.0 ± 0.00	N/A	0.301 ± 0.03	0.592 ± 0.06	0.793 ± 0.01	1.649 ± 0.03	N/E	0.005 ± 0.00	N/A	1.489 ± 0.02	1.545 ± 0.01	3.430 ± 0.01	N/E	N/E	0.016 ± 0.00	N/A
*E. coli*	8.5 ± 0.30	7.3 ± 0.25	4.5 ± 0.18	5.7 ± 0.25	N/E	18.0 ± 0.57	N/A	0.301 ± 0.03	0.592 ± 0.06	1.366 ± 0.16	1.649 ± 0.03	N/E	0.005 ± 0.00	N/A	1.489 ± 0.02	1.545 ± 0.01	3.430 ± 0.01	3.430 ± 0.01	N/E	0.005 ± 0.00	N/A
*E. faecalis*	9.6 ± 0.32	9.2 ± 0.20	5.7 ± 0.17	6.9 ± 0.10	N/E	22.0 ± 0.00	N/A	0.762 ± 0.02	0.592 ± 0.06	1.366 ± 0.16	1.649 ± 0.03	N/E	0.005 ± 0.00	N/A	1.489 ± 0.02	1.545 ± 0.01	3.430 ± 0.01	N/E	N/E	0.008 ± 0.00	N/A
*P. aeruginosa*	6.2 ± 0.29	5.9 ± 0.20	4.1 ± 0.10	N/E	N/E	24.0 ± 1.12	N/A	1.489 ± 0.02	1.545 ± 0.01	1.366 ± 0.16	N/E	N/E	0.005 ± 0.00	N/A	3.505 ± 0.01	3.642 ± 0.04	3.430 ± 0.01	N/E	N/E	0.012 ± 0.00	N/A
*S. aureus*	10.7 ± 0.30	10.2 ± 0.29	8.6 ± 0.21	7.5 ± 0.10	N/E	19.0 ± 1.22	N/A	0.301 ± 0.03	0.592 ± 0.06	0.793 ± 0.01	0.448 ± 0.03	N/E	0.001 ± 0.00	N/A	0.762 ± 0.02	1.545 ± 0.01	3.430 ± 0.01	1.649 ± 0.03	N/E	0.001 ± 0.00	N/A
*C. albicans*	8.1 ± 0.10	7.7 ± 0.12	7.2 ± 0.12	6.2 ± 0.25	N/E	N/A	22.0 ± 0.00	1.466 ± 0.02	1.532 ± 0.01	3.435 ± 0.01	1.635 ± 0.03	N/E	N/A	0.012 ± 0.00	3.517 ± 0.01	3.624 ± 0.04	3.435 ± 0.01	N/E	N/E	N/A	0.016 ± 0.00

MIC—minimum inhibitory concentration; MBC—minimum bactericidal concentration; MFC—minimum fungicidal concentration; N/E—no effect; N/A—not appliable. BL—basal leaves; CL—cauline leaves; ST—stems; BC—bracts; IF—inflorescences; TC—tetracycline; MC—miconazole. Values represent means of triplicate determinations (*n* = 3) ± standard deviations (*p* ≤ 0.05).

## Data Availability

Data are contained within the article.

## Supplementary Materials

The following supporting information can be downloaded at: https://www.mdpi.com/article/10.3390/antibiotics14121258/s1, File S1. Time-Kill Kinetics Assay Results of the Aerial Parts Extracts of *Cynara scolymus* L. File S2. Chromatograms of UV identification of phenolic compounds, without hydrolysis and after hydrolysis. Figure S1: HPLC chromatograms of the artichoke basal leaf extract; Figure S2. HPLC chromatograms of artichoke cauline leaf extract. Figure S3. HPLC chromatograms of artichoke stem extract. Figure S4. HPLC chromatograms of artichoke bract extract. Figure S5. HPLC chromatograms of artichoke inflorescence extract.
