# Supplementary material for: Antimicrobial Profile of Moldovan Cynara scolymus L.: Insights into Its Natural Antibiotic Potential
Source: Antibiotics (Basel). 2025 Dec 12;14(12):1258. doi: 10.3390/antibiotics14121258 (PMC12729854; doi:10.3390/antibiotics14121258)
Supplement: Supplementary file 1 [file antibiotics-14-01258-s001.zip › antibiotics-3991013-supplementary-HPLC-S2.pdf]

S2. Chromatograms of UV identification of phenolic compounds, without hydrolysis and after hydrolysis:

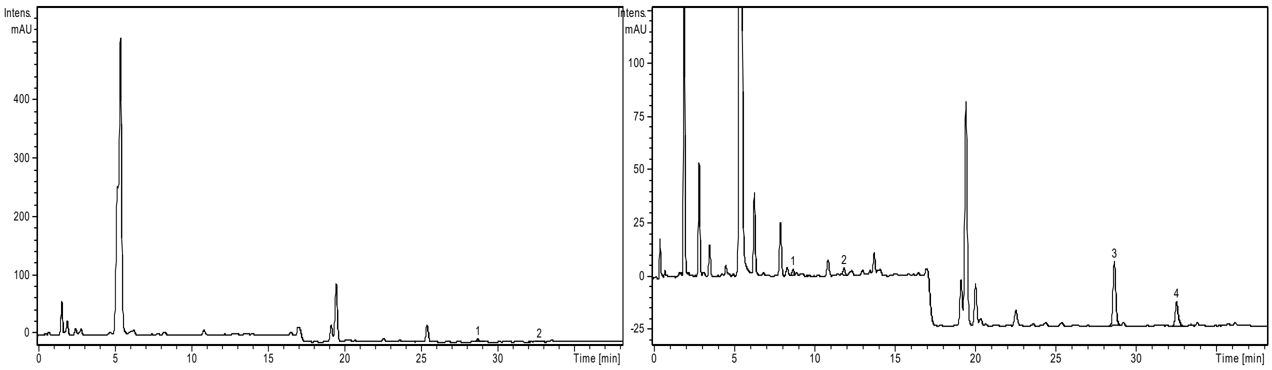

a. *b.*

Figure S1. HPLC chromatograms of the artichoke basal leaf extract.
(a. - without hydrolysis; b. - after acid hydrolysis).

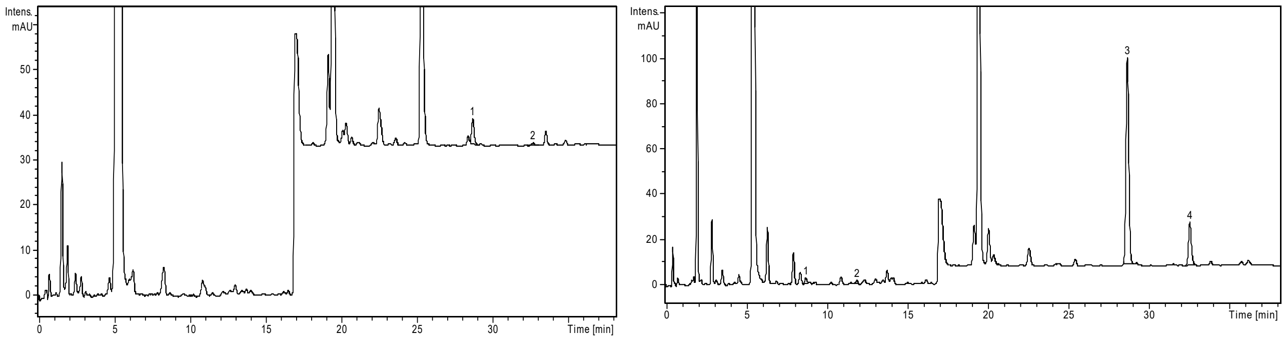

a. *b.*

Figure S2. HPLC chromatograms of artichoke cauline leaf extract.
(a. - without hydrolysis; b. - after acid hydrolysis)

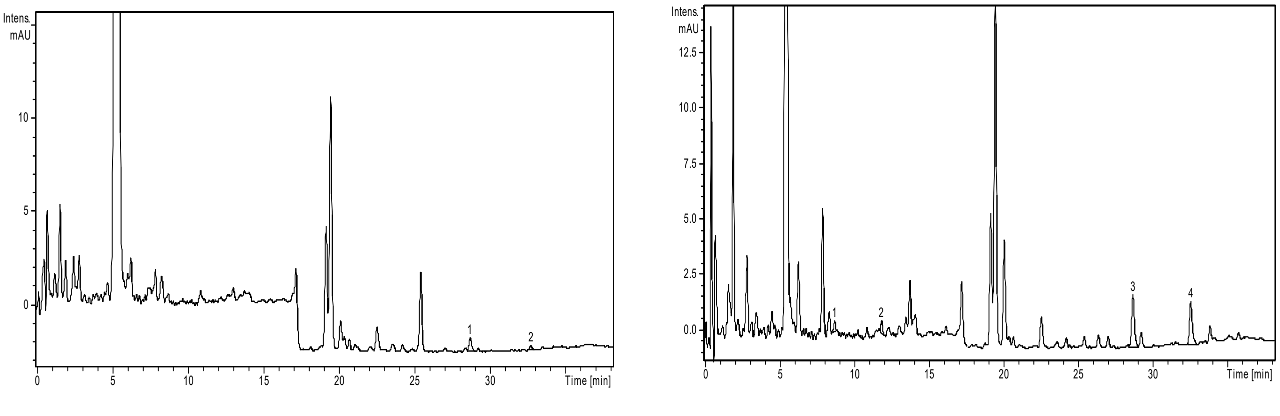

a. *b.*

Figure S3. HPLC chromatograms of artichoke stem extract.
(a. - without hydrolysis; b. - after acid hydrolysis)

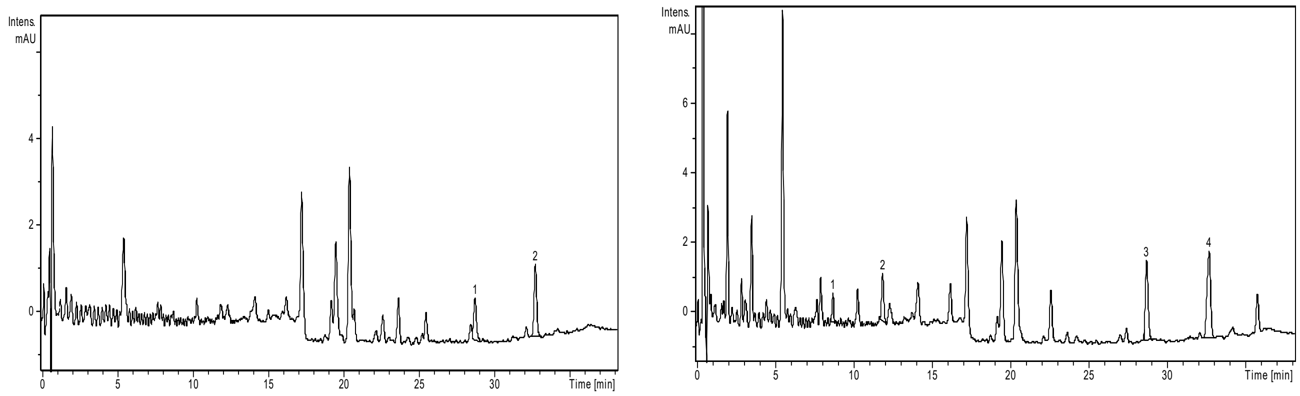

a. *b.*

Figure S4. HPLC chromatograms of artichoke bract extract.
(a. - without hydrolysis; b. - after acid hydrolysis)

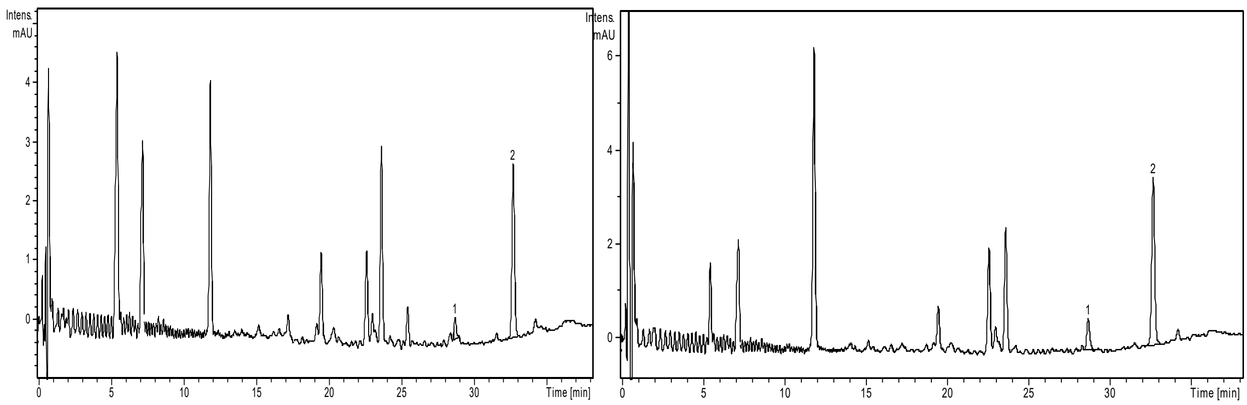

a. *b.*

Figure S5. HPLC chromatograms of artichoke inflorescence extract
(a. - without hydrolysis; b. - after acid hydrolysis)
