# Supplementary material for: Antimicrobial Profile of Moldovan Cynara scolymus L.: Insights into Its Natural Antibiotic Potential
Source: Antibiotics (Basel). 2025 Dec 12;14(12):1258. doi: 10.3390/antibiotics14121258 (PMC12729854; doi:10.3390/antibiotics14121258)
Supplement: Supplementary file 1 [file antibiotics-14-01258-s001.zip › antibiotics-3991013-supplementary-S1.pdf]

## S1. Time-Kill Kinetics Assay Results of the Aerial Parts Extracts of *Cynara scolymus* L.

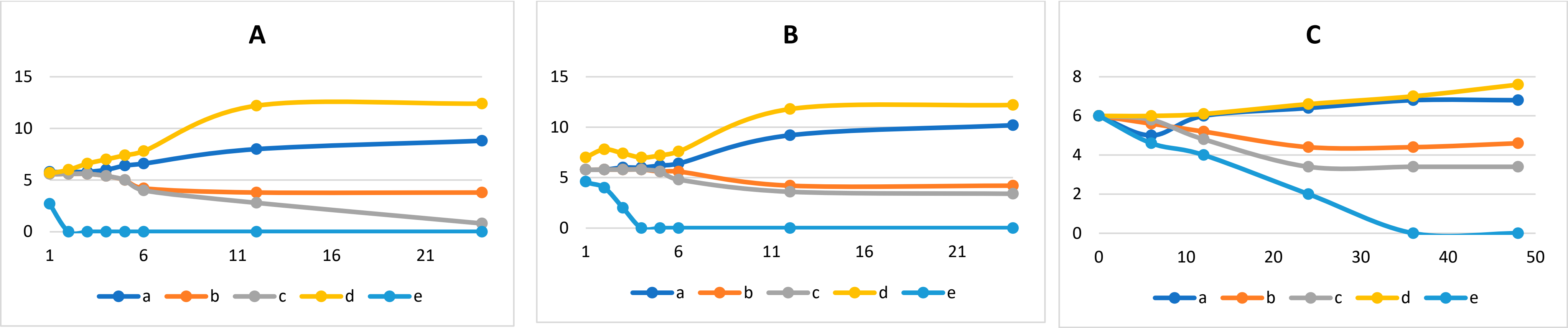


*In vitro* bacteriostatic and bactericidal activities of **basal leaves** extract against *S. aureus* ATCC 25923, MIC 0.301 mg/mL (A); *E. coli* ATCC 25922, MIC 0.301 mg/mL (B); *C. albicans* ATCC 10231, MIC 1.466 mg/mL (C) at the concentration of 0.5 x MIC (a), 1 x MIC (b), and 2 x MIC (c) compared with the untreated control (d) and tetracycline/miconazole (e). Data represent mean from three replicates.

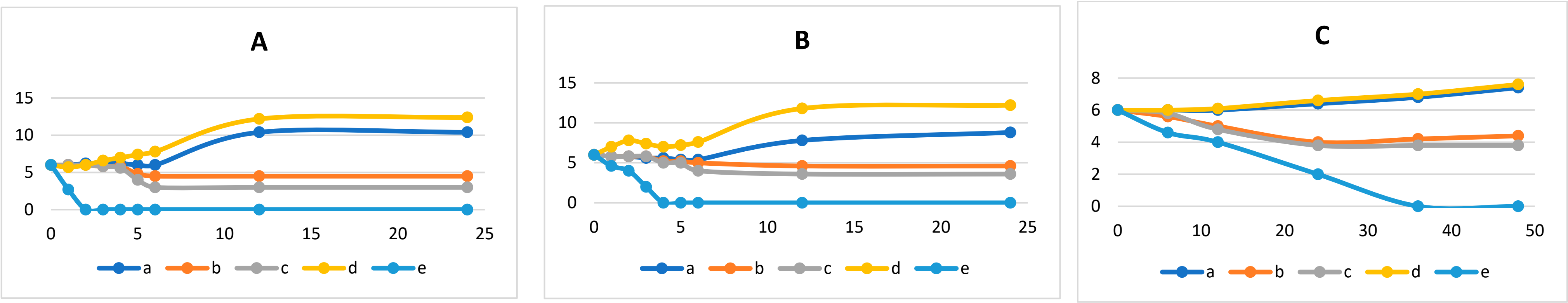


*In vitro* bacteriostatic and bactericidal activities of **cauline leaves extract** against *S. aureus* ATCC 25923, MIC 0.592 mg/mL (A); *E. coli* ATCC 25922, MIC 0.592 mg/mL (B); *C. albicans* ATCC 10231, MIC 1.532 mg/mL (C) at the concentration of 0.5 x MIC (a), 1 x MIC (b), and 2 x MIC (c) compared with the untreated control (d) and tetracycline/miconazole (e). Data represent mean from three replicates.

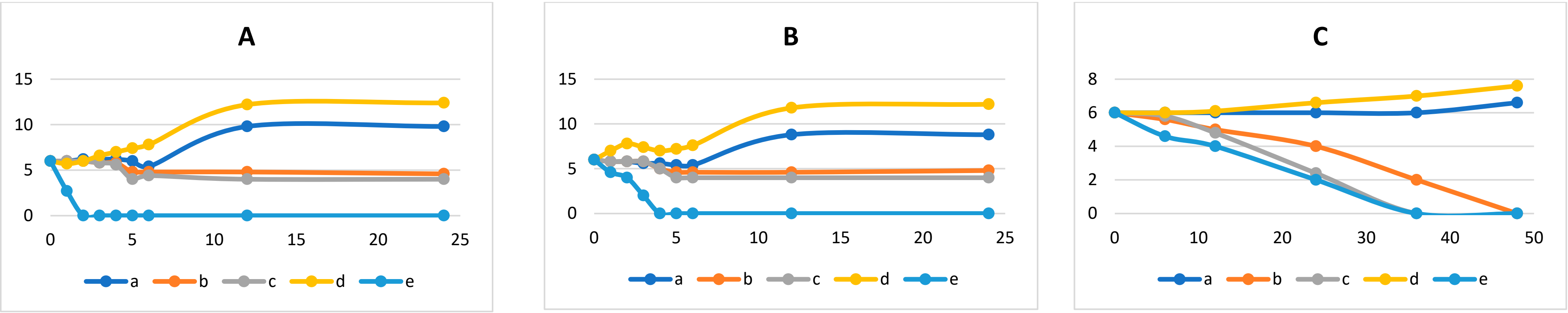


*In vitro* bacteriostatic and bactericidal activities of **stem extract** against *S. aureus* ATCC 25923, MIC 0.793 mg/mL (A); *E. coli* ATCC 25922, MIC 1.366 mg/mL (B); *C. albicans* ATCC 10231, MIC 3.435 mg/mL (C) at the concentration of 0.5 x MIC (a), 1 x MIC (b), and 2 x MIC (c) compared with the untreated control (d) and tetracycline/miconazole (e). Data represent mean from three replicates.

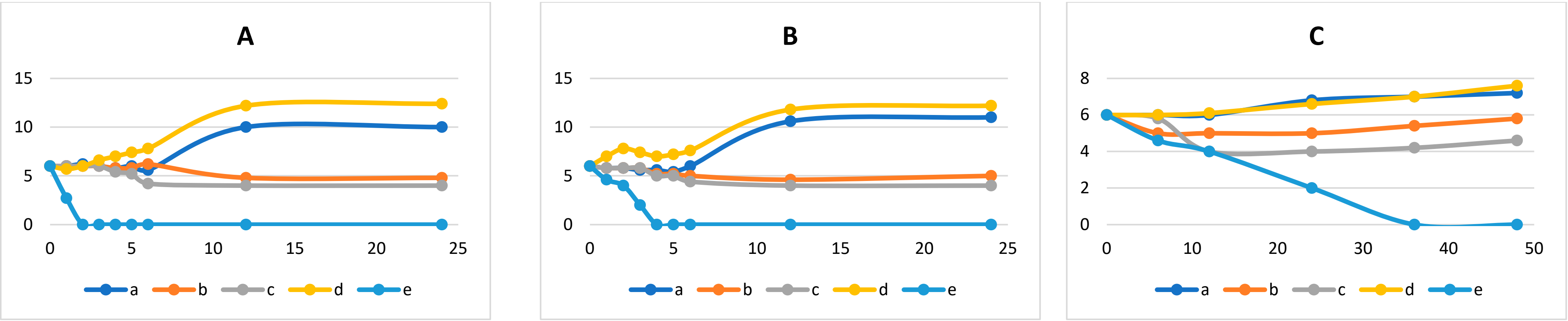


*In vitro* bacteriostatic and bactericidal activities of **bracts extract** against *S. aureus* ATCC 25923, MIC 0.448 mg/mL (A); *E. coli* ATCC 25922, MIC 1.649 mg/mL (B); *C. albicans* ATCC 10231, MIC 1.635 mg/mL (C) at the concentration of 0.5 x MIC (a), 1 x MIC (b), and 2 x MIC (c) compared with the untreated control (d) and tetracycline/miconazole (e). Data represent mean from three replicates.
